# Molecular characterization and correlation with β-lactam resistance of penicillin-binding protein2x, 2b, and 1a of *Streptococcus pneumoniae* in clinical pneumococcal isolates

**DOI:** 10.1128/spectrum.03850-25

**Published:** 2026-07-02

**Authors:** Youyi Zhang, Jiaming Shen, Feifei Hu, Biying Wang, ChangPeng Liu, Fan Jiang, Ran Peng, Xiaofei Liu, JingYuan Cheng, Chen Qian, Kaiyue Shen, Yingfeng Lu, LiPing Yi, Mengzhen Wang, Na He, Tao Zhang, Genming Zhao

**Affiliations:** 1 School of Public Health, Fudan University 12478, Shanghai, China; 2 Key Laboratory of Public Health Safety, Ministry of Education 659796, Shanghai, China; 3 Shanghai Institute of Infectious Disease and Biosecurity, Shanghai, China; 4 Zhangjiang Community Health Service Center, Shanghai, China; 5 Changzhou Center for Disease Control and Prevention, Jiangsu, China; University of Manitoba, Winnipeg, Manitoba, Canada

**Keywords:** *Streptococcus pneumoniae*, transpeptidases, penicillin-binding proteins, antibiotic resistance

## Abstract

Transpeptidases (TPDs), which belong to the penicillin-binding protein (PBP) families, are crucial for bacterial survival and are targeted by β-lactam antibiotics. We sought to determine the prevalence and positions of substitutions across the TPDs of the PBPs and their potential association with β-lactam resistance in pneumococcal strains. A total of 337 pneumococcal isolates were collected from pediatric patients in Suzhou, China, from 2013 to 2022. The serotype, antimicrobial susceptibility, and sequence in PBP1a, 2b, and 2x of these isolates were determined by Quellung reaction, E-test, and polymerase chain reaction sequencing, respectively. Homology modeling was utilized to compare the three-dimensional structure of the aberrant PBP strains with that of a reference R6 strain. Alterations within the PBPs conserved motifs and adjacent sequences SXXK and SXN contributed to penicillin resistance. Substitutions in TPDs were common and have a high degree of diversity. Two insertion amino acid (AA) motif variations, Tyr-Thr-Try(YTW) and Try-Thr-Tyr(WYT), located between AA 424 and 425 in PBP2b, were found to change the conformation of PBP2b in seven strains, and the YTW alteration has not been documented previously. The mutation rate of the TPDs in the PCV13 serotype strains was found to be higher than that observed in strains of non-vaccine serotypes. A high degree of diversity of TPD sequence in different PBPs was observed among the antibiotic non-susceptible pneumococcal isolates. The modeling analysis revealed conformational changes in the PBP2b following the insertion of the AA.

**IMPORTANCE:**

Since sequence variations both within and outside the penicillin-binding proteins have been reported to influence β-lactam minimum inhibitory concentrations (MICs), we examined the variability of isolates with transpeptidase (TPD) substitutions. Further quantification of the potential contributions of individual TPD positions and their interactions to β-lactam MICs could provide insights into resistance mechanisms and should be a priority of future investigation.

## INTRODUCTION

*Streptococcus pneumoniae* is an important global pathogen responsible for various infections, including bacterial pneumonia, sepsis, and meningitis (1, 2). β-Lactam antibiotics, such as penicillin and cephalosporin, are commonly recommended as first-line treatments for pneumococcal infections. However, since the first report of penicillin non-susceptible *S. pneumoniae* (PNSP) isolates in the 1960s (3), the prevalence of antibiotic-resistant pneumococci has steadily risen, becoming a significant public health concern (4, 5). β-Lactam antibiotics exert their bactericidal effects by inhibiting bacterial cell wall synthesis. They initiate the process through irreversible binding of the β-lactam ring to penicillin-binding proteins (PBPs) located on the bacterial cell membrane. Among these PBPs, transpeptidases are the most important components which play an essential role in the cross-linking of peptidoglycan, a polymer vital for maintaining cell wall structural integrity (6, 7). Therefore, alterations in the transpeptidase (TPD) domains of PBPs that reduce affinity for binding are primarily associated with the β-lactam resistance in clinical *S. pneumoniae* isolates, especially in PBP1a, PBP2b, and PBP2x (8–10).

Obviously, the TPD positions that exhibit frequent AA disparities between susceptible and resistant isolates appeared to be distributed nonrandomly. This may reflect structural constraints dictating that only alterations at specific TPD positions can give rise to viable, resistant PBP variants. Alternatively, it could suggest that the observed sequences of resistant TPDs have not yet diverged sufficiently from recent founder sequences. Additional research is needed to fully understand the relative contributions of these mechanisms (11). Additionally, there remains a substantial lack of data on PBP variants in *S. pneumoniae* isolated from mainland China (12, 13).

In this study, we investigated the mutations in the TPD region of PBPs in 337 *S. pneumoniae* isolates obtained from pediatric patients between 2013 and 2022 in Suzhou, China, and explored their association with β-lactam antibiotic resistance. The main purpose of this study was to explore the potential association between mutations in the TPD region of PBPs and β-lactam resistance in *S. pneumoniae*. By evaluating the prevalence of amino acid substitutions across the TPDs and pinpointing their positions through three-dimensional (3D) structural analysis exclusively for PBP2b, we aimed to elucidate the potential relationship between these genetic variations and the development of antibiotic resistance.

## MATERIALS AND METHODS

### Pneumococcal strains and serotyping

The *S. pneumoniae* strains analyzed in this study were isolated from children hospitalized at the Children’s Hospital of Soochow University (SCH) in Suzhou, China. To ensure accurate species identification, each strain was characterized using standard colonial morphology, Gram stain morphology, and the optochin susceptibility test. All isolates were stored in −80°C until further analysis.

Serotyping of *S. pneumoniae* was performed using the Quellung method with antiserum purchased from Statens Serum Institute (Copenhagen, Denmark). Initially, the serotype group was determined through a latex agglutination test employing a checkerboard typing system. A specific serotype antiserum was then mixed with the bacterial suspension, and the final serotype of pneumococci was confirmed by observing the swelling of the capsule under an oil immersion microscope. In cases where the test strain did not react with any of the antisera, it was classified as non-typeable (NT).

### Antibiotic susceptibility tests

Minimum inhibitory concentration (MIC) was determined by the E-test method for penicillin, ampicillin, chloramphenicol, clindamycin, cefotaxime, erythromycin, linezolid, levofloxacin, and moxifloxacin. *S. pneumoniae* ATCC49619 (China Center of Industrial Culture Collection, China) was used as the quality control strain. Antimicrobial susceptibility testing results were interpreted according to Clinical Laboratory Standard Institute guidelines from 2019 (https://clsi.org/) (14).

### Sequencing of PBP genes

Pneumococci were cultivated in plates containing 5% defibrinated sheep blood at 37°C with 5% CO_2_. Bacterial genomic DNA was extracted using the BD MagNA Pure 96 DNA and Viral NA Small Volume Kit. The extracted DNA was stored at −20°C until use. To amplify partial fragments of *pbp1a*, *pbp2b*, and *pbp2x* genes by polymerase chain reaction, we used three sets of primers, as described in Table 1. The amplified fragments (0-based amino acid coordinates) positions 161–710 (549 AA) of the 719AA full-length PBP1a, 219–659 (440 AA) of the 680AA full-length PBP2b, and 209–665 (456 AA) of the 750AA full-length PBP2x (Fig. S1).

**TABLE 1:** Primer sequences of *PBP1a*, *PBP2x*, and *PBP2b*

Gene targeted	Primer	Primer sequence (5′–3′)	Product size (bp)
*PBP1a*	AF	CGGCATTCGATTTGATTCGCTTCT	2,120
	AR	GTCGTACTATTATTTGTGCTTGGAGTGGTT	
*PBP2x*	XF	CCGGAGTAAGATATGAAGTGGACAAAAAGAGTAATCCG	2,123
	XR	AACATATTAGTCTCCTAAAGTTAATTTAATTTTTTTAATG	
*PBP2b*	BF	GATCCTCTAAATGATTCTCAGGTGGCTGT	1,321
	BR	GTCAATTAGCTTAGCAATAGGTGTTGGAT	

Bidirectional sequencing and sequence stitching are performed in the BDA Sequencing Company. The spliced sequences were translated into simulated protein sequences and compared with PBP2x, PBP2b, and PBP1a corresponding to the reference strains of *S. pneumoniae* R6 (GenBank entry number: NC003098) for mutation analysis.

### 3D structure prediction

To elucidate the probable locations of AA substitutions within the PBPs’ structure, we conducted a 3D structure of the PBP2b with AA substitutions and insertion mutation using homology modeling. The crystal structure of the PBP2b sequence from *S. pneumoniae* R6 was obtained from the Protein Data Bank (PBP2b PDB ID: 2WAF). For homology modeling, we utilized the automated mode of the SWISS-MODEL Repository (https://swissmodel.expasy.org/). Chimera 1.18 (https://www.cgl.ucsf.edu/chimera/download.html), a molecular structure visualization software developed by the Resource for Biocomputing, Visualization, and Informatics (RBVI) at the University of California, San Francisco, was employed for the analysis of the 3D structure data (15).

### Statistical analysis

Sequences of the *pbp* genes were edited, aligned, and subjected to nucleotide and protein analysis using MEGA version 11. Sequences were trimmed to cover the active-site motifs of the TPDs, with precise 0-based amino acid ranges of 371–647 for PBP1a, 379–656 for PBP2b, and 229–587 for PBP2x (Fig. S1). R version 4.3.2 software was used for statistical analysis, and graphics were also created in R version 4.3.2. The Pearson chi-square test/Fisher’s exact test and the *t*-test were used to compare the differences in categorical variables between groups. A two-sided *P* value of less than 0.05 was considered statistically significant.

## RESULTS

### Demographic features and antibiotic resistance patterns of *S. pneumoniae* isolates

Among all the strains collected, a total of 337 clinical pneumococcal strains were prospectively collected (Table 2). Most of the strains (97.03%) were isolated from non-invasive specimens, including sputum (*n* = 175, 51.93%) and ear discharge (*n* = 152, 45.10%), while the remaining 10 strains were isolated from invasive specimens, comprising cerebrospinal fluid (*n* = 5, 1.48%), blood (*n* = 4, 1.19%), and bronchoalveolar lavage fluid (*n* = 1, 0.30%). Among the strains, four were non-typeable, while the most prevalent serotype was 19F (*n* = 163, 48.37%), followed by 19A (*n* = 42, 12.46%), 6B (*n* = 24, 7.12%), 6A (*n* = 21, 6.23%), 23F (*n* = 18, 5.34%), and 14 (*n* = 16, 4.75%). After the introduction of the coverage of 13-valent Pneumococcal Conjugate Vaccine (PCV13) in Suzhou (2018-2022), there was a significant increase in the proportion of non-vaccine serotypes (NVTs) compared to the pre-PCV13 period (2013–2017) (18.00% vs 5.84%, *P* = 0.002).

**TABLE 2:** Demographics and characteristics of *S. pneumoniae* isolates

Characteristics		Total			2013-2017			2018-2022		*χ* ^2^	*P* value
		*N*	%		*N*	%		*N*	%		
Gender											
	Male	206	61.13		85	62.04		121	60.50	0.030	0.864
	Female	131	38.87		52	37.96		79	39.50		
Age											
	0–< 5	309	91.69		131	95.62		178	89.00	3.849	0.050
	5–17	28	8.31		6	4.38		22	11.00		
Serotype^*a*^											
	VT	293	86.94		129	94.16		164	82.00	9.548	0.002
	NVT	44	13.06		8	5.84		36	18.00		
PEN											
	S	252	74.78		88	64.23		164	82.00	42.614	<0.001
	I	63	18.69		47	34.31		16	8.00		
	R	22	6.53		2	1.46		20	10.00		
AMX											
	S	142	44.24		49	35.77		93	50.54	7.272	0.122
	I	102	31.78		52	37.96		50	27.17		
	R	77	23.99		36	26.28		41	22.28		
CTX											
	S	152	47.06		50	37.59		102	53.68	9.789	0.044
	I	66	20.43		28	21.05		38	20.00		
	R	105	32.51		55	41.35		50	26.32		

^*a*^ VT (PCV13 vaccine serotype): 1, 3, 4, 5, 6A, 6B, 7F, 9V, 14, 18C, 19A, 19F, 23F; NVT (non-vaccine serotype): 23A, 35F, 15A, 15B; untypeable: 19B, 35B, 6C, 13, 6D, 36, 39, 42, 10F, 11C, 15F, 19C; PEN, penicillin; AMX, amoxicillin; CTX, cefotaxime; S, antibiotic-susceptible *S. pneumoniae*; R, antibiotic-resistant *S. pneumoniae*; I, antibiotic-intermediate *S. pneumoniae*.

During the study period, the antibiotic non-susceptibility rates were as follows: 25.22% for penicillin, 55.77% for amoxicillin, and 52.94% for cefotaxime. Notably, after the introduction of the PCV13, reductions in non-susceptibility rates were observed for penicillin (35.77% vs 18.00%, *P* < 0.001) and cefotaxime (62.40% vs 46.32%, *P* = 0.044).

### Association between penicillin susceptibility and variations in PBPs conserved sites

Sequence variations in the conserved motifs SXXK, SXN, and KT(S)G of PBPs were analyzed in 337 *S. pneumoniae* isolates, revealing alterations in these motifs and adjacent sequences contributing to the antibiotic resistance of penicillin. There were no changes in the conserved motifs of SRN and KTG in PBP1a for penicillin-susceptible *S. pneumoniae* (PSSP) and PNSP, while 98.82% of PNSP and 84.13% of PSSP had T371A (Thr→Ala) or S (Thr→Ser) substitutions in the STMK motif, a difference that was statistically highly significant (*P* < 0.001, Fisher’s exact test). In the PBP2b conserved motifs or nearby sites, 98.82% of PNSP and 90.87% of PSSP had T446A substitution (Thr→Ala) or S (Thr→Ser) substitutions in the SSNT motif, a difference that was statistically significant (*P* = 0.024, Fisher’s exact test). In contrast, no alterations were detected in the other two PBP2b motifs. In the PBP2x conserved motifs or nearby sites, 87.30% of PSSP and 95.29% of PNSP had T338A substitution (Thr→Ala) or K (Thr→Lys), with a statistically significant between-group difference (*P* = 0.021, Fisher’s exact test). Meanwhile, a statistically significant difference in the Met339Phe substitution of the STMK motif was observed between PSSP and PNSP (39.29% vs 55.29%, *P* = 0.011, Fisher’s exact test). And 95.29% of PNSP and 86.90% of PSST had L546V substitution (Leu→Val) or D (Leu→Asp) in the LKSG motif (Table 3).

**TABLE 3:** Association of penicillin susceptibility and PBP active binding sites’ motif alterations

Motif	Variants	PSSP (*n* = 252)	PNSP (*n* = 85)	*P* value^*a*^
PBP1a				
STMK	SSMK	178 (70.63)	77 (90.59)	<0.001
	SAMK	34 (13.49)	7 (8.24)	
	–^*c*^	40 (15.87)	1 (1.18)	
SRN	–	252 (100.00)	85 (100.00)	
KTG	–	252 (100.00)	85 (100.00)	
PBP2b				
SVVK	–	252 (100.00)	85 (100.00)	
SSNT^*b*^	SSNA	223 (88.49)	82 (96.47)	0.024
	SSNS	6 (2.38)	2 (2.35)	
	–	23 (9.13)	1 (1.18)	
KTG	–	252 (100.00)	85 (100.00)	
PBP2x				
STMK	SAMK	120 (47.62)	34 (40.00)	0.021
	SAFK	99 (39.29)	47 (55.29)	
	SKMK	1 (0.40)	0 (0.00)	
	–	32 (12.70)	4 (4.71)	
HSSN^*b*^	LSSN	10 (3.97)	1 (1.18)	0.303
	–	242 (96.03)	84 (98.82)	
LKSG^*b*^	DKSG	3 (1.19)	1 (1.18)	0.076
	VKSG	216 (85.71)	80 (94.12)	
	–	33 (13.10)	4 (4.71)	

^*a*^ *P* values were calculated using two-sided Fisher’s exact test.

^*b*^ The adjacent residues analyzed here refer to His394 in the 394–397 HSSN motif of PBP2x, Leu546 in the 546–549 LKSG motif of PBP2x, and Thr446 in the 443–446 SSNT motif of PBP2b.

^*c*^ '–’, no data.

### Correlation between TPD domains variants of PBPs and antibiotic resistance

**Fig 1:**
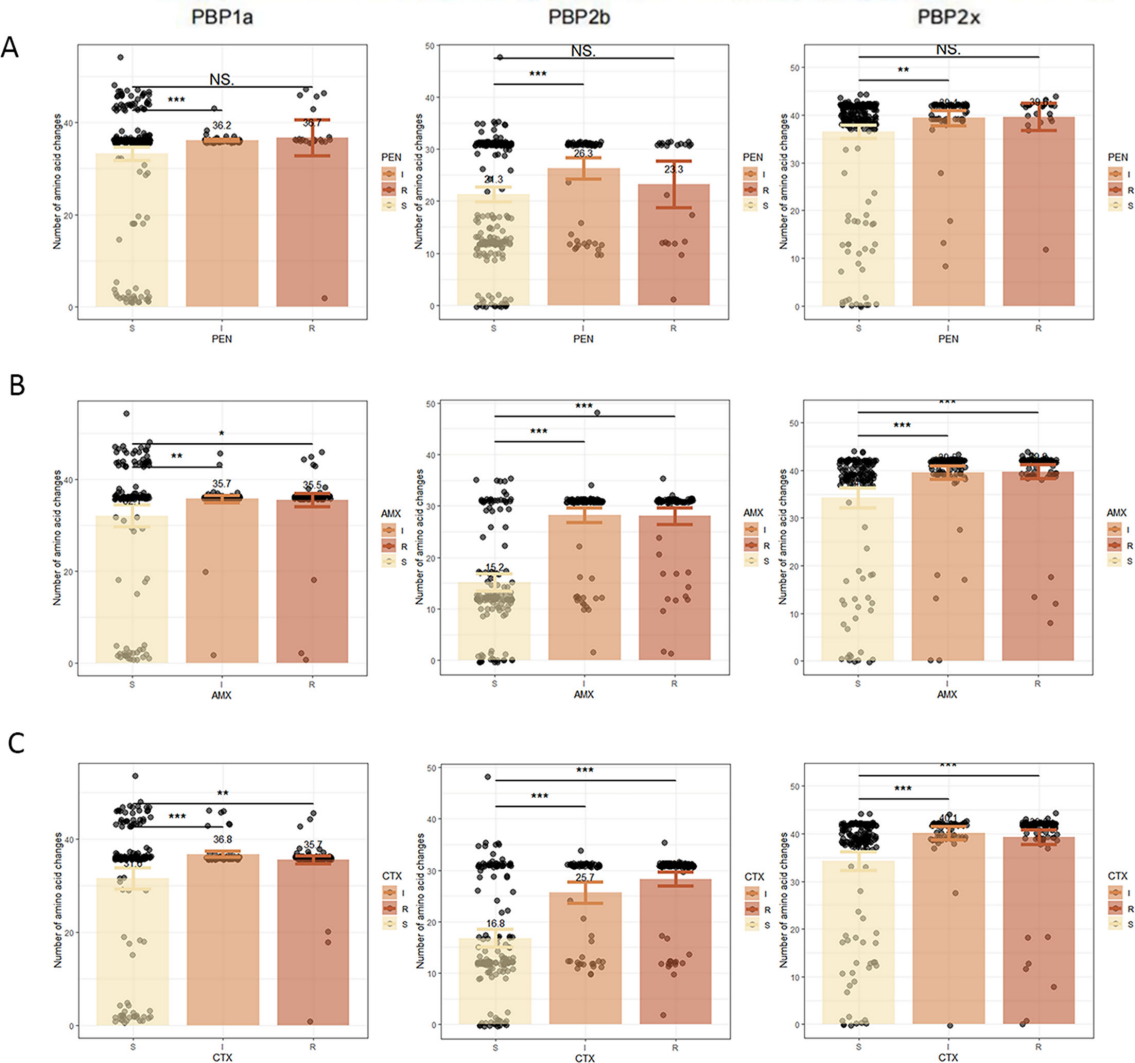
β-Lactam antibiotics MICs increased with the AA differences of the TPDs of PBPs. (**A**) Penicillin MICs increased with the number of AA change of the TPDs of PBP1a, PBP2b, and PBP2x. (**B**) Amoxicillin MICs increased with the number of AA change of the TPDs of PBP1a, PBP2b, and PBP2x. (**C**) Ceftriaxone MICs increased with the number of AA change of the TPDs of PBP1a, PBP2b, and PBP2x. Each individual circle denotes a single *S. pneumoniae* isolates. The value bars with NS are not significant, asterisks denote statistically significant differences. *, *P* < 0.05; **, *P* < 0.01; ***, *P* < 0.001.

The three TPD domains collectively comprised 914 AA sites. The AA sequence identities of the TPDs domains of PBP1a, 2b, and 2x genes from the 337 *S. pneumoniae* strains ranged from 80.51% to 99.64%, 82.80% to 100.00%, and 80.65% to 100.00%, respectively, with overall similarity between 98.7% and 99.9%, compared to the same three genes in GenBank (accession no. NC003098). Our study revealed that the MICs of penicillin, amoxicillin, and ceftriaxone increased with the total number of divergences in the TPDs of PBP2x, PBP1a, and PBP2b (Fig. 1).

### Association of TPD mutations with antimicrobial resistance and vaccine serotype profiles

**Fig 2:**
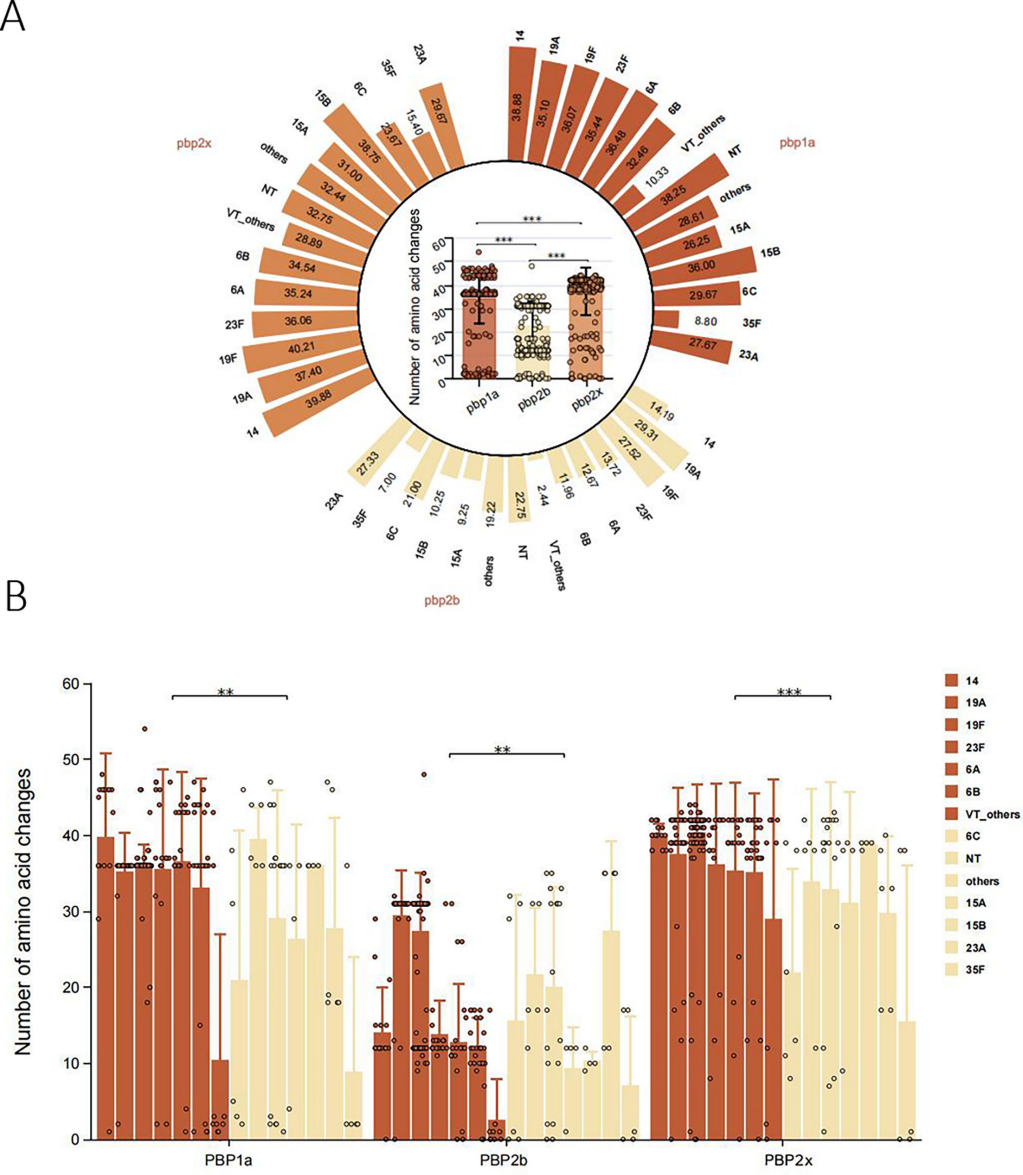
The relationship between TPD alterations and penicillin susceptibility of common serotypes. (**A**) AA mutation rate of the TPDs of PBPs in different serotypes. Each individual circle denotes a single *S. pneumoniae* isolates; non-typeable (NT). (**B**) AA mutation rate of the TPDs of PBPs in different vaccine serotypes. The orange column is the PCV13-serotype strains, and the light yellow is non-vaccine serotypes strains. Each individual circle denotes a single *S. pneumoniae* isolates; non-typeable (NT); PCV13 vaccine serotype (VT); VT_others: 9V, 3, 4, 5, 7F, 18C.

**Fig 3:**
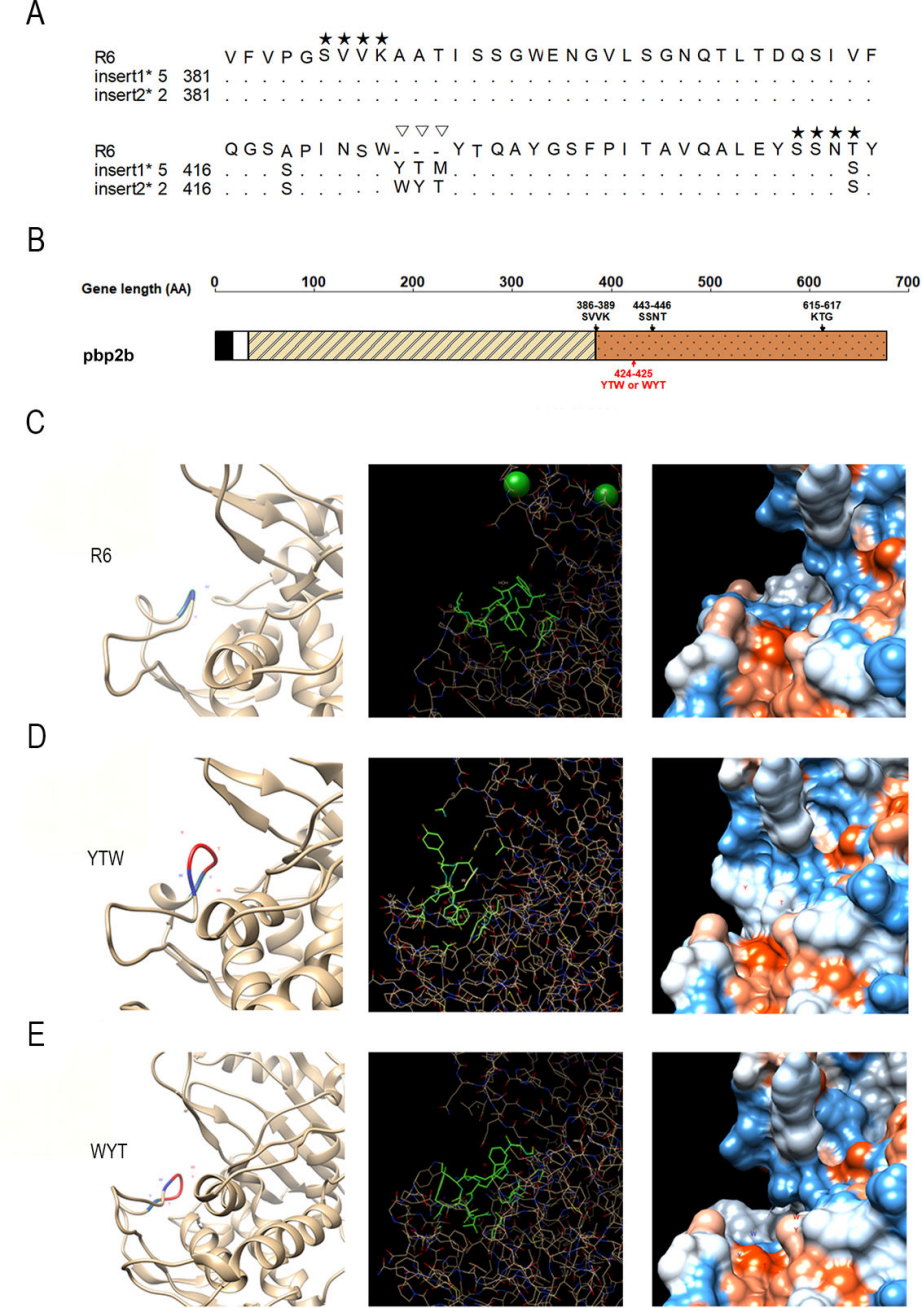
Schematic representation of the inserted AA residue location in the pbp2b gene and the three-dimensional structure of the PBP2b insertion site region. (**A**) Alignment of AA sequences of the TP domains from residues 380 to 447 of PBP2b from clinical isolates and the R6 strain, with the insertion site at positions 424–425 highlighted. Pentagrams indicate core conserved motifs and their immediately adjacent functional site, and triangles mark the positions of inserted AA residues. (**B**) Schematic representation of the corresponding location of inserted AA residues at the pbp2b gene. The native PBP2b*R6 protein. Solid and empty boxes denote the cytoplasmic domain and membrane anchor, respectively. Within the periplasmic region, the N-terminal domain is shown as a yellow hatched box, and the TP domain as an orange-red box. Conserved motifs and their immediately adjacent are labeled above the schematic, with the AA insertion site marked below using red text and arrows. (**C**) The 3D structures of PBP2b from *S. pneumoniae* R6 (PDB ID: 2WAF). (**D**) The 3D of YTW insertion on the PBP2b gene. (**E**) The 3D of WYT insertion on the PBP2b gene. In the protein ribbons structure, ribbons W424 and Y425 in medium blue, WYT and YTW in red, and KSG in yellow. In the all atoms protein structure, marked <5 angstroms from the insertion site region atoms. In the hydrophobicity surface, blue represents hydrophilic areas, and orange represents hydrophobic areas.

To achieve a detailed and comprehensive characterization of mutation distribution along the TPDs gene, we calculated the percentage of AA substitution for every five consecutive AA residues by comparing with the reference strain R6 (GenBank accession no. NC003098). A total of 112 AA mutations were detected in the pbp2x gene, while 75 different substitutions and two insertion variations were detected in the PBP2b sequence. Eighty-three AA mutations were detected in the pbp1a gene (Fig. S2). The insertion variations were only found in seven strains (2.08%, 7/337), located between AA 424–425 in PBP2b, and the insertion sequences were YTW (1.33%, 5/337) and WYT (0.59%, 2/337) (Fig. 3A and B). The AA mutation rate in the TPD region of PBP1a and pbp2x was higher than that of pbp2b (<0.001) (Fig. 2A).

AA substitutions of TPDs of PBPs in PCV13-serotype strains were higher than non-vaccine serotypes (*P* < 0.01). Serotypes 14 and 19F exhibited the highest frequency of substitutions in the pbp2x fragments, while serotypes 14 and NT strains showed high substitutions in pbp1a, and serotypes 19A and 19F had high mutation rates of AA in pbp2b (Fig. 2B).

### The TPDs AA insertion of *S. pneumoniae* PBP2b mapped against the crystal structure of *S. pneumoniae* PBP2b

Swiss-Model homology modeling was employed to simulate the 3D impact of YTW and WYT insertion on the PBP2b gene by reference strain R6. The modeling analysis revealed conformational changes in the PBPs following the insertion of the amino acid sequences into the TPDs of PBP2b. The serotypes of the seven strains exhibiting AA insertions were identified as follows: five strains were identified as serotype 6B, one strain was identified as serotype 6D, and one strain was identified as serotype 19F. Notably, all these strains were discovered as post-2018 isolates (Fig. 3C through E).

## DISCUSSION

Understanding the mechanisms of antibiotic resistance in bacteria is crucial. In *S. pneumoniae*, resistance to β-lactam antibiotics primarily involves modifications to PBPs. PBPs are key enzymes that catalyze the synthesis of peptidoglycan in the bacterial cell wall and are also the target of β-lactam antibiotics (16). Variations across the whole TPDs of PBPs have been associated with penicillin resistance (11, 17).

This study integrated gene mutation analysis with protein modeling techniques to elucidate the effects of mutations on the structure of PBPs. Our study found that over 91.30% of antibiotic-resistant *S. pneumoniae* exhibited variations in PBP1a at positions T371A or T371S without alterations in the conserved motifs of SRN and KTG. These motifs have been associated with high-level resistance to penicillin and cephalosporins, as previously reported in multinational studies (18). Furthermore, over 95.65% of antibiotic-resistant *S. pneumoniae* strains had T446A or T446S substitutions in the SSNT motif, with no changes observed in the other two PBP2b motifs, consistent with previous findings (19, 20). Common substitutions in PBP2x active sites included T338A, M339F, H394L, and L546V, aligning with earlier studies (21). Therefore, amino acid changes in the conserved motifs of *S. pneumoniae* PBPs correlated with altered resistance to β-lactam antibiotics.

AA substitutions resulting from nucleotide mutations in three PBPs were observed across the entire TPDs, with the highest frequency in PBP2X (112 AA mutations), followed by PBP1A (83 AA mutations) and PBP2B (75 AA mutations). Significant increases in AA substitutions of three PBPs were observed in 14, 19A, and 19F. Malawi studies have reported that pneumococcal serotypes 6A, 13, 14, 16F, 19A, 19F, 23F, and 35B, which are the highest-ranking serotypes for acquired pbp fragments, are associated with higher MICs for penicillin compared to those without acquired fragments (22). Notably, the *pbp2x* and *pbp1a* genes associated with β-lactam resistance are positioned at both ends of the cps locus. The evolution of penicillin resistance through capsular switching is likely to continue in *S. pneumoniae* strains not covered by the current vaccines, due to selection pressures arising from the widespread pneumococcal vaccination and the overuse of antibiotics. Therefore, ongoing high-quality molecular epidemiological surveillance is imperative.

We also identified two insertion mutations, YTW and WYT, in seven strains, located between AA 424 and 425 in the active site of PBP2b. Five strains were identified as serotype 6B, one strain was identified as serotype 6D, and one strain was identified as serotype 19F. The WYT insertion has previously been reported in 1996 in Japan, suggesting a possible shared evolutionary pathway or selective advantage (19). Notably, one of these insertions—the YTW variant—has not been previously documented. Similarly, Chinese studies have reported two insertion variations, YIW and YTW, between positions 386SVVK and 443SSN in the active site of PBP2b (the two strains were of serotype 6B and NT) (21). It seems that the serotype 6 strain exhibits a higher susceptibility. According to our homology modeling, the insertion of AA may alter the protein’s conformation, particularly because the insertion site is proximal to key SVVK and SSN motifs. Of note, the insertion site 424–425 is less than 20 AAs (17 sites) away from the conserved site of SSNT (443–446). Li et al. observed a modest yet significantly higher density of such positions within 20 AAs from a TPD active site (11).

The insertion at positions 424–425 identified in this study is located within the same pre-α4 loop segment as the resistance-associated mutation sites N422Y, T426K, and Q427L reported in the literature and represents an AA sequence alteration event within this resistance hotspot region. This region is a key gatekeeper region at the entrance of the PBP2b active site (23). While some of the positions have been implicated in altering affinity to β-lactams, many others were of unclear function (23). Therefore, we hypothesized that amino acid insertion mutations, particularly those located near conserved sequences, would gradually influence the resistance of *S. pneumoniae*. However, further studies are needed to confirm their function and impact.

In this study, the most prevalent serotypes were 19F (48.37%), 19A (12.46%), 6B (7.12%), 6A (6.23%), 23F (5.34%), and 14 (4.75%). The observed serotype distribution is basically consistent with findings from other Chinese studies. We observed that, after the introduction of the PCV13 in Suzhou, there was a significant increase in the proportion of NVT compared to the pre-PCV13 period. Notably, reductions in resistance rates were observed for penicillin, amoxicillin, and cefotaxime. The mutation rate of the TPDs in the PCV13 serotype strains was found to be higher than that observed in strains of NVT. Similar results have been documented in other countries. A Japanese study reported a significant reduction in penicillin resistance genotypes decreasing from 54.3% to 11.2%, and the observed decline in both PRSP and PISP strains was associated with a reduction in strains of the PCV13 serotypes (24). Previous researchers observed that the implementation of PCVs has led to a notable decline in the overall prevalence of antibiotic resistance in pneumococci. However, significant increases in resistance to penicillin and erythromycin were observed among NVT isolates (25). In summary, we observed that the introduction and vaccination of the vaccine caused a decrease in VT and an increase in NVT, leading to a decrease in antibiotic resistance in the short term. However, vaccination records for these patients were not collected, and the direct effect of the vaccine could not be explained. We hypothesize that the observed decline in antibiotic resistance is temporary, and the ongoing use of antibiotics may facilitate the acquisition of antibiotic resistance genes over time. Consequently, we anticipate an eventual increase in drug resistance rates.

Previous studies have demonstrated that sequence variations both within and outside the PBPs can significantly influence β-lactam MICs (26). Therefore, we examined the sequence variability of isolates with TPD substitutions. Future work should prioritize quantifying the individual TPD positions and their interactions to β-lactam MICs, which will advance our understanding of the underlying resistance mechanisms.

## Data Availability

The raw sequence data reported in this paper have been deposited in the National Center for Biotechnology Information at PV208010-PV208046, PV274066-PV274223, and PV394276-PV394605.

## SUPPLEMENTAL MATERIAL

Fig. S1 spectrum.03850-25-s0001.tif Schematic representation of the gene and TPDs of PBP1a, PBP2b, and PBP2x.

Fig. S2 spectrum.03850-25-s0002.tif Heatmap of 5-AA mutation rate in TPD region and penicillin susceptibility.

Supplemental legends spectrum.03850-25-s0003.docx Descriptive legends for Fig. S1 and S2.

ASM does not own the copyrights to Supplemental Material that may be linked to, or accessed through, an article. The authors have granted ASM a non-exclusive, world-wide license to publish the Supplemental Material files. Please contact the corresponding author directly for reuse.
