# Supplementary material for: Molecular characterization and correlation with β-lactam resistance of penicillin-binding protein2x, 2b, and 1a of Streptococcus pneumoniae in clinical pneumococcal isolates
Source: Microbiol Spectr. 2026 Jul 2;14(8):e03850-25. doi: 10.1128/spectrum.03850-25 (PMC13436343; doi:10.1128/spectrum.03850-25)
Supplement: Supplementary file 1 [file spectrum.03850-25-s0003.docx]

**Supplementary Legends**

Supplementary Figure 1. Schematic representation of the gene and TPDs of PBP1a, PBP2b and PBP2x

The x-axis represents the amino acid (AA) position of the full-length coding sequence, with a scale marked at 100 AA intervals. For each PBP-encoding gene, three regions are visualized as colored bars: the full-length coding sequence (Total Gene), the PCR-amplified fragment (PCR segment), and the transpeptidase domain. Black arrows and labels indicate the position of conserved active-site motifs critical for PBP enzymatic activity within the transpeptidase domain. Cream indicates the full-length coding sequence (CDS) of the gene; burnt orange indicates the PCR-amplified fragment; copper indicates the transpeptidase domain.

Supplementary Figure 2. Heatmap of 5-AA mutation rate in TPD region and penicillin susceptibility**.**

(A) PBP1a alternation rates’ heat map among PSSP and penicillin-non-susceptible *S. pneumoniae* PNSP; (B) PBP2b alternation rates’ heat map among PSSP and PNSP; (C)PBP2x alternation rates’ heat map among PSSP and PNSP. Each column corresponds to an individual strain, with 'S' indicating pen-sensitive strains and 'I/R' indicating pen-insensitive strains; Each row, from top to bottom, represents the mutation rate for every five amino acids within the TPD region
